# Antioxidant and Anti-Inflammatory Effect of Probiotic *Lactobacillus plantarum *KU15149 Derived from Korean Homemade Diced-Radish Kimchi

**DOI:** 10.4014/jmb.2002.02052

**Published:** 2020-03-24

**Authors:** Kyoung Jun Han, Ji-Eun Lee, Na-Kyoung Lee, Hyun-Dong Paik

**Affiliations:** Department of Food Science and Biotechnology of Animal Resources, Konkuk University, Seoul 05029, Republic of Korea

**Keywords:** Probiotics, kimchi, *Lactobacillus plantarum*, antioxidant, anti-inflammatory

## Abstract

*Lactobacillus plantarum* KU15149 was demonstrated to have probiotic behavior and functions, including antioxidant and anti-inflammatory activity. *L. plantarum* KU15149 obtained from homemade diced-radish kimchi has a high survival rate under artificial gastric acid (pH 2.5, 0.3% pepsin) and bile salt (0.3% oxgall) conditions. However, *L. plantarum* KU15149 did not produce β-glucuronidase, which is known to be a carcinogenic enzyme with resistance to several antibiotics, such as gentamycin, kanamycin, streptomycin, tetracycline, and ciprofloxacin. *L. plantarum* KU15149 strongly adhered to HT-29 cells and had high antioxidant activity in terms of 2,2-diphenyl-1-picrylhydrazyl (DPPH) free radical-scavenging and β-carotene bleaching assays. *L. plantarum* KU15149 also exhibited a pronounced inhibition of nitric oxide (NO) production, along with expression of nitric oxide synthase (*iNOS*) and cyclooxygenase -2 (*COX-2*) as well as pro-inflammatory cytokines, such as *TNF-α*, *IL-1β*, and *IL-6*, when RAW 264.7 cells were stimulated with LPS. Therefore, *L. plantarum* KU15149 exhibited pharmaceutical functionality as a potential probiotic.

## Introduction

Oxidative stress is caused by a pro-oxidant and antioxidant imbalance that leads to the generation of toxic reactive oxygen species (ROS), such as hydrogen peroxide, organic hydroperoxides, superoxide, hydroxyl radicals, and nitric oxide. Persistent oxidative damage of tissue and cellular components can cause several diseases and accelerate the aging process in humans [1].

Inflammatory reactions and ROS perform indispensable physiological functions in immune defense and cell signaling [2]. However, undue or continued ROS production and inflammation may result in a number of health problems, such as type 2 diabetes, cardiovascular disease, osteoporosis, insulin resistance, inflammatory bowel disease, arthritis, and asthma [3]. Therefore, ROS levels and inflammatory responses are important for reducing the risk of related chronic diseases.

The inflammatory response in the body is a defense against risk stimuli, including microbial infections, endotoxins, and tissue damage, and it is necessary to restore the normal structure and function of tissues. Normal inflammatory responses have a regulatory process during which the production of pro-inflammatory mediators decreases over time, while anti-inflammatory mediators rise in number, thereby limiting the inflammatory response itself [4]. Macrophages, one of the cell types involved in the body’s inflammatory response, play an important role in this inflammatory response. Macrophages are activated by pro-inflammatory cytokines, such as *tumor necrosis factor (TNF)-α, interleukin (IL)-1β, IL-6,* and lipopolysaccharide (LPS), and exposure to LPS stimulation, which is a bacterial cell membrane component. In addition, macrophages produce various inflammatory mediators, such as nitric oxide (NO) through expression of inducible nitric oxide synthase (*iNOS*) and cyclooxygenase -2 (*COX-2*) [5].

Probiotics are defined as live microorganisms that when administered in appropriate amounts, confer a beneficial effect upon the host [6] and contribute to the regulation of immune responses [7]. Lactic acid bacteria (LAB) have been used as probiotics and can survive under strong acid and bile salt conditions while adhering to the cells of the intestinal tract [8]. LAB strains are also generally known to be non-pathogenic and sensitive to antibiotics [9]. In addition, several studies have reported that probiotics have antimicrobial, antioxidant, anticancer, antiallergy, and immune-enhancing activities [10,11]. Probiotics have also been reported to have several positive and beneficial effects on human health [12].

Kimchi is a Korean traditional fermented vegetable product that is gaining popularity as a functional food as it contains various LAB, such as *Lactobacillus plantarum* and *Leuconostoc mesenteroides* [13]. In particular, LAB strains isolated from kimchi can survive in the intestines due to their strong resistance to acid and bile salt conditions [14]. Kimchi LAB are probiotic strains that exert various beneficial effects, such as antioxidant, antimicrobial, antimutagenic, anticancer, immune-regulation, anti-inflammatory, antiallergic, anti-obesity, cholesterol-lowering, and lipid-lowering activities [15].

*L. plantarum* is the most important and prominent microorganism involved in the middle and latter steps of kimchi fermentation [16]. *L. plantarum* is a generally recognized as safe (GRAS) microorganism and has been used as a probiotic strain and fermentation starter in various foods, including meat, dairy products, plant-based products, and coffee [17]. Furthermore, various studies have reported that *L. plantarum* has immune stimulation, inflammatory reduction, and antioxidant activities [18].

Therefore, the aim of the present study was to determine the probiotic properties, safety, and functional effects of LAB strains isolated from homemade diced-radish kimchi. Further, the antioxidant and anti-inflammatory activities of the isolated strains were investigated.

## Material and Methods

### Chemicals and Reagents

MRS and oxgall were purchased from Becton Dickinson Biosciences (USA). Pepsin, ascorbic acid, β-carotene, linoleic acid, chloroform, Tween 80, Triton X-100, 2,2-diphenyl-1-picrylhydrazyl (DPPH), lipopolysaccharide (LPS), 3-(4,5-dimethylthiazol-2-yl)-2,5-diphenyltetrazolium bromide (MTT), dimethyl sulfoxide (DMSO), and Griess reagent were purchased from Sigma Chemical Co., Ltd. (USA). Dulbecco’s Modified Eagle’s Medium (DMEM), RPMI1640, water, antibiotics, fetal bovine serum (FBS), phosphate-buffered saline (PBS), and 1% streptomycin/penicillin solution were purchased from HyClone Laboratories, Inc. (USA). The API ZYM kit was purchased from bioMérieux (France). Specific primers used for performing the real-time polymerase chain reaction (RT-PCR) were purchased from Bionics (Korea).

### Bacterial Strains and Sample Preparations

*Lactobacillus plantarum* KU15149 was isolated from homemade diced-radish kimchi. Kimchi samples (1 g) were diluted and inoculated on MRS agar at 37°C for 24 h. The selected strain was inoculated and incubated in MRS broth at 37°C for 24 h and identified as *L. plantarum* by 16S rRNA gene sequencing. The commercial probiotic strain, *L. rhamnosus* GG (LGG), was obtained from the Korean Collection for Type Cultures (Korea) and used as a reference probiotic strain. To harvest the LAB cells, bacterial cell cultures were centrifuged at 12,000 ×*g* for 10 min and washed three times with PBS. The LAB cells were suspended in PBS.

### Cell Cultures

HT-29 (human colon adenocarcinoma) and RAW 264.7 (murine macrophage) cell lines were obtained from the Korean Cell Line Bank (Korea) and respectively cultured in RPMI and DMEM supplemented with 10% FBS and 1% streptomycin/penicillin solution at 37°C in a humidified atmosphere containing 5% CO_2_.

### Tolerance to Artificial Gastric Conditions of LAB Strains

The tolerance of LAB strains to artificial gastric conditions was evaluated as described by Lee *et al*. [19]. The LAB strains were incubated in an overnight culture and resuspended in the MRS medium (pH 2.5) containing 0.3% (w/v) pepsin and oxgall, and then incubated at 37°C for 3 h and 24 h. The number of surviving bacteria was determined by counting viable cells on the MRS plates.

### Enzyme Production of LAB Strains

Enzyme production was assessed using the API ZYM kit. The LAB strains were centrifuged at 12,000 ×*g* for 10 min, and the cell pellet was resuspended in PBS at 10^5^ CFU/ml and added to the cultures. After inoculation, the mixtures were incubated at 37°C for 4 h and reagents zym A and zym B were each added. The level of enzyme activity was determined as 0 (no activity) to 5 (≥ 40 nM) based on the color change.

### Adhesion of LAB Strains to HT-29 Cells

The adhesion ability of LAB strains to HT-29 cells was described as the percentage of viable bacteria remaining as compared to the initial bacterial counts added. Adherence to HT-29 cells of LAB strains was assessed by the methodology of Son *et al*. [11]. For the adhesion assays, 1 × 10^5^ cells/ml of HT-29 cells were seeded onto a 24-well cell culture plate and incubated at 37°C for 24 h. The LAB strains were centrifuged at 12,000 ×*g* for 10 min and washed twice with PBS before being inoculated on the wells at approximately 10^7^ CFU/ml and incubated for 2 h at 37°C. Non-adhesive bacteria were removed by washing three times with PBS. Next, 1 ml of 1% (v/v) Triton X-100 was added into each well and incubated for 10 min at 37°C. Following incubation, the cells were separated from the wells. The number of adherent bacterial cells was indicated by counting viable cells on the MRS plates.

### Antibiotic Sensitivity of LAB Strains

Sensitivity of the LAB strains was measured according to the guidelines of the Clinical and Laboratory Standards Institute (CLSI) [20]. The disc diffusion method was applied to determine sensitivity to clinical antibiotics, such as ciprofloxacin (5 mg), gentamicin (10 mg), ampicillin (10 mg), streptomycin (10 mg), tetracycline (30 mg), kanamycin (30 mg), doxycycline (30 mg), and chloramphenicol (30 mg). Each LAB strain, at a concentration of 10^7^ CFU/ml, was spread on MRS agar, and paper discs containing the antibiotics were placed on plates after a few minutes. Subsequent to incubation for 24 h at 37°C, the diameters of the inhibition zones were measured.

### Preparation of Bacterial Cells

The LAB strains were grown in MRS broth overnight, centrifuged at 12,000 ×*g* for 10 min, and washed twice with PBS. The washed bacterial cells were resuspended in PBS to a final concentration of 10^8^ CFU/ml.

### Free Radical-Scavenging Activity toward DPPH of the LAB Strains

Antioxidant activity was determined by DPPH free radical-scavenging activity according to Yang *et al*.’s method [21]. A volume of 0.4 mM of DPPH solution was prepared in methanol, and 2 ml of bacterial cells or distilled water (control) were mixed with the same volume of this solution. The mixtures were incubated for 30 min at room temperature in the dark. The absorbance of the supernatants was measured at 517 nm after centrifugation at 12,000 ×*g* for 10 min, and calculated as follows:


$$\text{DPPH radical scavenging activity (\%)}=(1–\frac{A_{\mathrm{sample}}}{A_{\mathrm{control}}})\times 100$$

### β-Carotene Bleaching Assay

A β-carotene bleaching assay was conducted as described by Kachouri *et al*. [22] with some modifications. β-carotene solution was prepared by mixing 3 mg of β-carotene, linoleic acid (66 μl), and Tween 80 (300 μl) with chloroform (10 ml). Chloroform was then removed in a rotary evaporator at 40°C under vacuum and the remaining solution was diluted with 100 ml of distilled water. For the assay, 200 μl of samples or distilled water (control) was mixed with 4 ml of this solution and incubated in a water bath at 50°C for 2 h. Next, absorbance was measured at 470 nm for 0 and 2 h, and inhibition activity was calculated as follows:


$$\text{Inhibition activity of β-carotene oxidation (\%)}=(\frac{A_{\mathrm{sample},2h}–A_{\mathrm{control},2h}}{A_{\mathrm{sample},0h}–A_{\mathrm{control},0h}})\times 100$$

### Cell Viability of RAW 264.7 Cells by LAB Strains

The effect of LAB strains on the viability of RAW 264.7 cells was evaluated using the MTT assay according to the method described by Han *et al*. [23] with some modifications. RAW 264.7 cells (1 × 10^6^ cells/ml) were plated on 96-well cell plates for 24 h. Next, LAB strains were treated with 10^7^ CFU/ml and incubated for 44 h. After aspiration of supernatant for determination, cells were treated with MTT solution (2.5 mg/ml in PBS) and incubated for 4 h. Upon discarding the supernatant, DMSO was added to each well and the generated formazan deposits were dissolved. Absorbance of each well was measured at 570 nm using a microplate reader. Cell viability was calculated as a percentage of the absorbance.

### NO Production in RAW 264.7 Cells

The production of NO in LPS-induced RAW 264.7 cells was assessed according to the methods of Lee *et al*. [19]. RAW 264.7 cells (2 × 10^5^ cells/well) were plated in 96-well plates and treated with LAB strains (10^7^ CFU/ml) and LPS (1 μg/ml) for 24 h. The samples were centrifuged at 12,000 ×*g* for 1 min after incubation, and 100 μl of the supernatants was added to the same amount of Griess reagent. Absorbance of the mixtures was assessed at 540 nm using a microplate reader. NO production was calculated through comparison with the standard curve constructed with sodium nitrate as a standard.

### Anti-Inflammatory Effect of LAB Strains

The anti-inflammatory effect of LAB strains was measured as described by Lee *et al*. [19] and Son *et al*. [24]. RAW 264.7 cells were seeded on a 6-well plate (1 × 10^6^ cells/ml) and incubated for 24 h and then added into LPS-stimulated (1 μg/ml) and LAB strains (10^7^ CFU/ml). RNA was isolated from RAW 264.7 cells treated with samples using the RNeasy Mini Kit (QIAGEN) and cDNA was synthesized using the Revert Aid First Strand cDNA Synthesis Kit (Thermo Fisher Scientific). The expression levels of iNOS and cytokines related to anti-inflammatory effects were measured using synthesized cDNA as a template and a PCR mixture containing SYBR Green PCR Master Mix using RT-PCR (PikoReal 96, Thermo Scientific Pierce). RT-PCR was performed as per the following conditions: 95°C for 2 min followed by 40 cycles of 95°C for 5 sec and 60°C for 15 sec. The results were analyzed after normalization with *β-actin* as a reference gene and calculated using the 2^–ΔΔCt^ method. The melting curve analysis was performed to assess reaction specificity. The primer sequences are listed in Table 1.

### Statistical Analysis

All experiments were repeated in triplicate and presented as the mean ± standard deviation. One-way analysis of variance (ANOVA) and Duncan’s multiple range test were applied to determine the degree of significant differences. Values were considered significant at *p* < 0.05, and all analyses were conducted with the Statistical Package for the Social Sciences (SPSS), version 24 (IBM, USA) program.

## Results and Discussions

### Tolerance to Gastric Conditions of LAB Strains

Tolerance to artificial gastric conditions are characteristic of LAB strains required for probiotic properties in the intestine [25]. The tolerance of *L. rhamnosus* GG, *L. plantarum* KU15149 and *L. brevis* KU15176 to artificial gastric acids and bile salts is indicated in Table 2. All LAB strains exhibited high resistance at 0.2 log CFU to artificial gastric conditions, including pH 2.5 and 0.3% pepsin. *L. rhamnosus* GG exhibited the highest resistance to 0.3% oxgall. The viable cell counts of *L. plantarum* KU15149 and *L. brevis* KU15176 were slightly decreased by 1.53 and 0.92 log CFU with bile salts, respectively. *L. plantarum* Lb41 isolated from kimchi was reduced by 0.06 log CFU/ml and 1.36 log CFU/ml under strongly acidic and bile salt conditions, respectively [17]. Therefore, *L. plantarum* KU15149 and *L. brevis* KU15176 possessed probiotic properties due to resistance to artificial gastric conditions, and these strains could be expected to survive in the gastrointestinal tract.

### Enzymatic Activities of LAB Strains

Certain probiotic bacteria are of value as they express enzymes such as α-glucosidase, β-glucosidase, and β-galactosidase. β-galactosidase hydrolyzes lactose into glucose and galactose in milk and alleviates the lactose intolerance problem experienced by some adults [26, 27]. In addition, probiotic bacteria should not produce enzymes such as β-glucuronidase, which has been associated with the induction of carcinogenesis, mutagens, and toxins [28]. The enzymatic activities of the LAB strains tested are shown in Table 3. Although *L. rhamnosus* GG, *L. plantarum* KU15149, and *L. brevis* KU15176 produced β-galactosidase, none of the LAB strains produced β-glucuronidase. A previous study indicated that probiotic *L. plantarum* FI10604 and *L. brevis* FI10700 also do not produce β-glucuronidase [11]. *Lactococcus lactis* KC24 produces various enzymes, such as acid phosphatase, β-galactosidase, and naphthol-AS-BI-phosphohydrolase, but does not produce β-glucuronidase [19].

### Antibiotic Sensitivity of LAB Strains

The sensitivity of probiotic bacteria to antibiotics is a fundamental precondition because antibiotic-resistant strains may not be easily eliminated if required, and their antibiotic resistance may be transmitted to pathogenic or potentially pathogenic bacteria [29]. The antibiotic sensitivity of LAB strains is presented in Table 4. *L. brevis* KU15176 had the same sensitivity as *L. rhamnosus* GG, and in the case of *L. plantarum* KU15149, it had a similar sensitivity, except to tetracycline (30 mg) and chloramphenicol (30 mg). *L. plantarum* Ln4 has been shown to be sensitive to commercial antibiotics, such as ampicillin, chloramphenicol, doxycycline, and tetracycline [11]. Therefore, these results confirm that LAB strains are safe in accordance with the CLSI guidelines.

### Adhesion of LAB Strains to HT-29 Cells

**Fig. 1:**
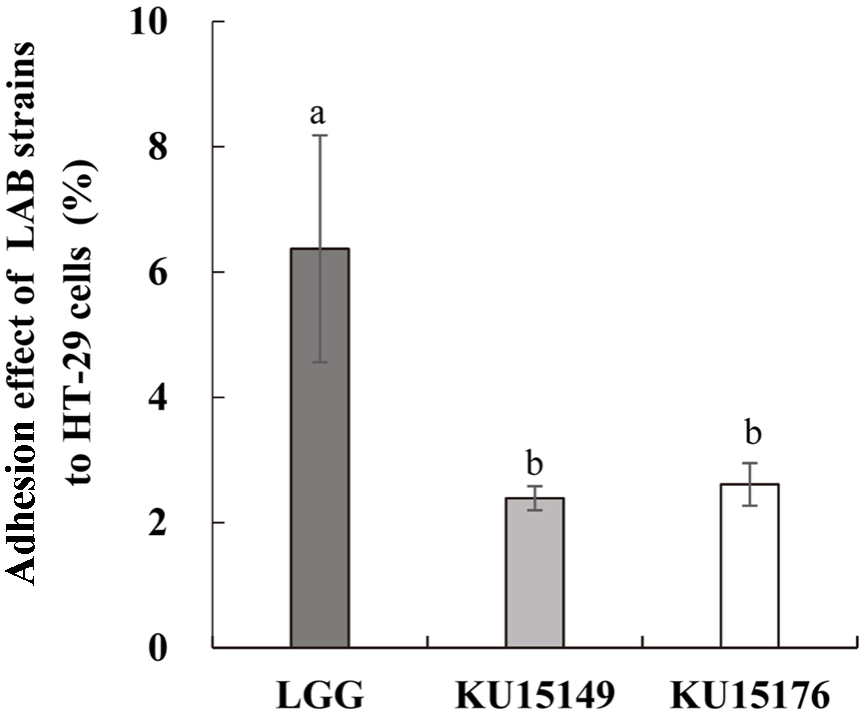
Adhesion of LAB strains to HT-29 cells. LGG, *L. rhamnosus* GG; KU15149, *L. plantarum* KU15149; KU15176, *L. brevis* KU15176. Error bars indicate standard deviation of three independent experiments. Letters indicate a significant difference between prospective and commercial probiotic strain. All values are expressed as the mean ± standard deviation. Values with different letters indicate significant differences for each characteristic (*p* < 0.05).

The adhesion of LAB strains to intestinal cells is the most important factor in their effective use [30]. LAB strain adhesion to HT-29 cells is shown in Fig. 1. *L. plantarum* KU15149 and *L. brevis* KU15176 demonstrated a similar ability to adhere to HT-29 cells. *L. rhamnosus* GG had the greatest adhesion ability (6.37%), followed by *L. plantarum* KU15149 (2.39%) and *L. brevis* KU15176 (2.61%). Song *et al*. [31] previously showed that *L. brevis* KCCM 12203P (6.84%) has a slightly higher adhesion ability to intestinal epithelial cells compared to that of *L. rhamnosus* GG (6.21%). Zhang *et al*. [32] showed that *L. plantarum* C32, C42, and C62 (< 2%) have diminished adhesion ability to intestinal epithelial cells compared to that of *L. rhamnosus* GG (4.30%). Therefore, although the adhesion ability of *L. plantarum* KU15149 (2.39%) and *L. brevis* KU15176 (2.61%) to intestinal cells was lower than that of the commercial strain, *L. rhamnosus* GG (6.21%), this was still considered sufficient for use as a functional probiotic.

### Antioxidant Effect of LAB Strains

**Fig. 2:**
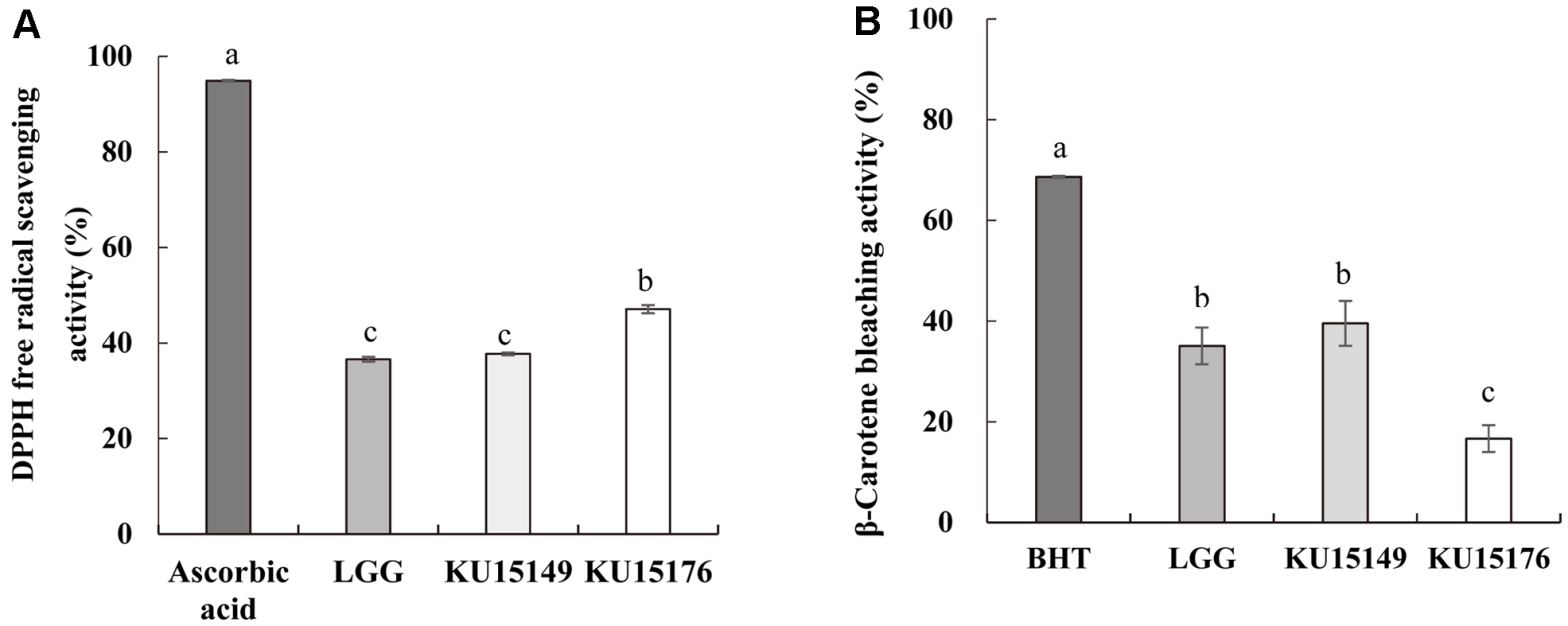
Antioxidant activity of LAB strains assessed by (A) DPPH free-radical scavenging and (B) β-carotene bleaching assays. BHT, Butylated hydroxytoluene; LGG, *L. rhamnosus* GG; KU15149, *L. plantarum* KU15149; KU15176, *L. brevis* KU15176. Letters a–c denote a significant difference between prospective and commercial probiotic strains. Each value is expressed as the mean ± standard deviation. The different letters on the error bars represent statistically significant differences between values (*p* < 0.05).

The critical role of LAB strains in antioxidant activity is protection from free radicals [33]. The antioxidant activity of LAB strains was assessed by DPPH free radical scavenging (Fig. 2A) and β-carotene bleaching assays (Fig. 2B). *L. brevis* KU15176 (47.09%) showed significantly higher DPPH free-radical scavenging activity than that of *L. rhamnosus* GG (36.60%), while the activity of *L. plantarum* KU15149 (37.73%) was similar to that of *L. rhamnosus* GG. In a prior study, *L. brevis* KU15153 (44.14%) has been shown to exhibit higher DPPH free-radical scavenging activity than *L. rhamnosus* GG (19.21%) [34]. The β-carotene bleaching inhibition activity of LAB strains is depicted in Fig. 2B. The β-carotene bleaching inhibition activity of *L. plantarum* KU15149 (39.57%) was higher than that of *L. brevis* KU15176 (16.65%) but not significantly different from that of *L. rhamnosus* GG (35.08%). Another study found that *L. paraplantarum* SC61 showed 35.64% inhibition of β-carotene bleaching activity [24].

### Anti-Inflammatory Activity of LAB Strains

To confirm cytotoxicity in RAW 264.7 cells, the viability of LAB was determined using the MTT assay (data not shown). The LAB strains were shown to have higher viability at 10^7^ CFU/ml than at 10^8^ CFU/ml. Therefore, LAB strains were tested at a concentration of 10^7^ CFU/ml for induction of NO synthesis in macrophages.

**Fig. 3:**
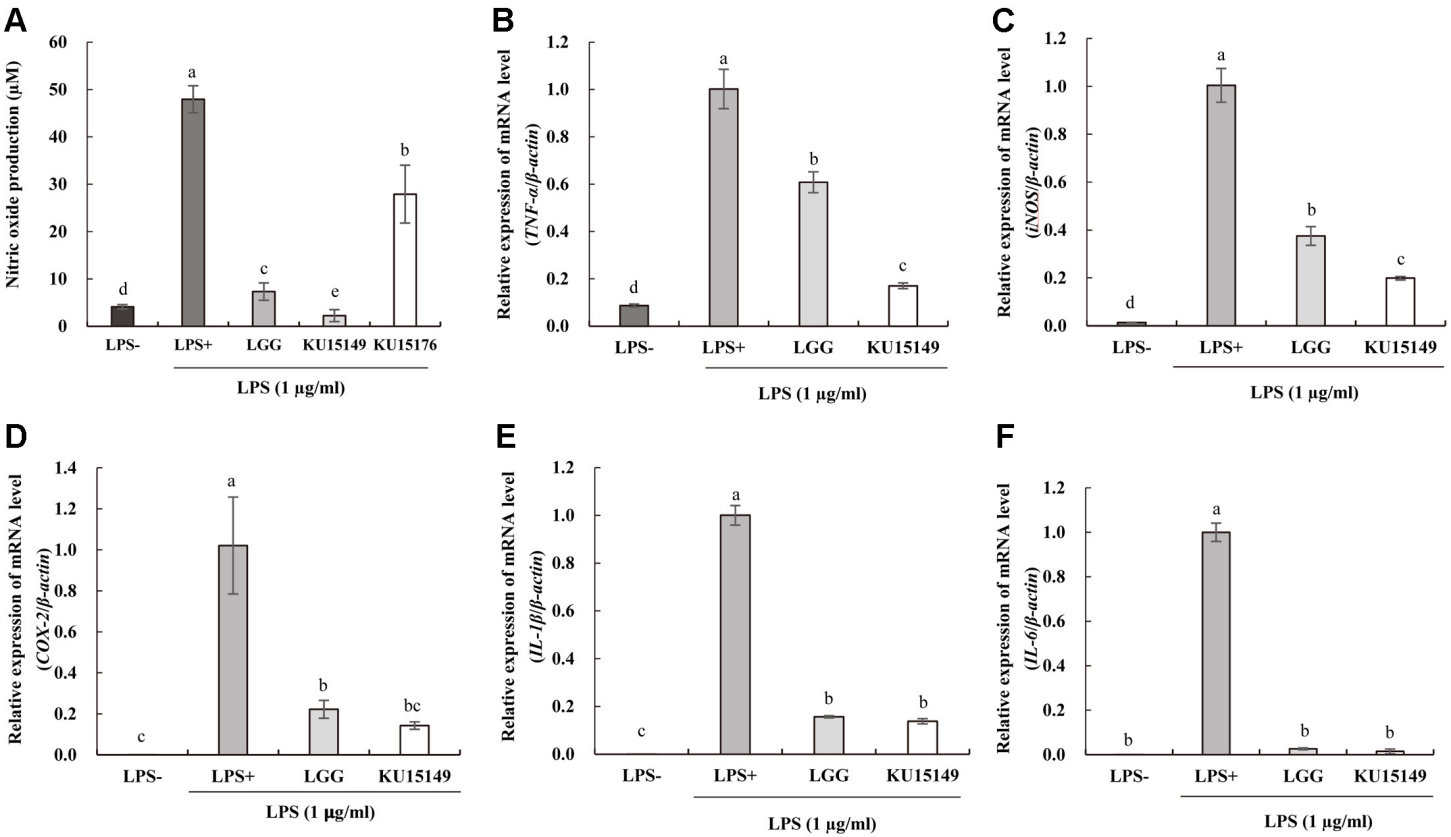
Production of (A) NO on LAB strains in lipopolysaccharide (LPS)-stimulated RAW264.7 cells and the relative expression of mRNA level of (B) *TNF-α*, (C) *iNOS*, (D) *COX-2,* (E) *IL-1β*, and (F) *IL-6*. LPS-, without LPS treatment; LPS+, treated with LPS (1 µg/ml); LGG, *L. rhamnosus* GG with LPS; KU15149, *L. plantarum* KU15149 with LPS. All values are expressed as the mean ± standard deviation and standardized against the *β-actin* housekeeping gene. Values with different letters indicate significant differences for each characteristic (*p* < 0.05).

The NO-producing activity of *L. plantarum* KU15149 (2.25 μM) was the lowest in comparison to that of *L. rhamnosus* GG (7.32 μM) and *L. brevis* KU15176 (25.57 μM) in stimulating LPS conditions (Fig. 3A). *Lactococcus lactis* NK34 has been reported to possess low levels of NO production [23] and *L. plantarum* 4B15 and 4M13 have been found to exert the greatest inhibitory effect on NO production [35].

LAB strains were analyzed for mRNA expression levels of *TNF-α*, *iNOS*, *COX-2*, *IL-1β*, and *IL-6* from RAW 264.7 cells through RT-qPCR (Figs. 3B-3F). Both *L. plantarum* KU15149 and *L. rhamnosus* GG demonstrated significant decrease in all five mRNAs assessed when compared to the LPS+ control. *L. plantarum* KU15149 was associated with a significant decrease in relative expression of *TNF-α* and *iNOS* in comparison to *L. rhamnosus* GG, suggesting that *L. plantarum* KU15149 would be more effective in dampening an LPS-induced immune response; levels of *COX-2*, *IL-1β*, and *IL-6* mRNA expression showed no significant difference between the two strains. *Weissella cibaria* JW15 has been shown to significantly inhibit the expression of pro-inflammatory cytokines compared to *L. rhamnosus* GG [36].

In conclusion, *L. plantarum* KU15149 isolated from homemade diced-radish kimchi was found to have probiotic properties including gastric acid and bile salt tolerance, enzyme activity, adhesion to intestinal cells, and antibiotic sensitivity. *L. plantarum* KU15149 had a high abundance of antioxidants, as measured by DPPH free-radical scavenging and β-carotene bleaching inhibition. In addition, *L. plantarum* KU15149 induced lower levels of NO and less expression of pro-inflammatory cytokine genes *TNF-α* and *iNOS* than did *L. rhamnosus* GG under LPS-induced conditions in macrophages. Therefore, *L. plantarum* KU15149 could be potentially used as a probiotic and anti-inflammatory ingredient.

## Figures and Tables

**Table 1:** Primer sequences related to anti-inflammatory effect used in real-time PCR.

Primer^a^		Primer sequence (5’–3’)
TNF-α	Sense	5’-TTG ACC TCA GCG CTG AGT TG-3’
	Antisense	5’-CCT GTA GCC CAC GTC GTA GC-3’
iNOS	Sense	5’-CCC TTC CGA AGT TTC TGG CAG CAG C-3’
	Antisense	5’-GGC TGT CAG AGC CTC GTG GCT TTG G-3’
COX-2	Sense	5’-CAC TAC ATC CTG ACC CAC TT-3’
	Antisense	5’-ATG CTC CTG CTT GAG TAT GT-3’
IL-1β	Sense	5’-CAG GAT GAG GAC ATG AGC ACC-3’
	Antisense	5’-CTC TGC AGA CTC AAA CTC CAC-3’
IL-6	Sense	5’-GTA CTC CAG AAG ACC AGA GG-3’
	Antisense	5’-TGC TGG TGA CAA CCA CGG CC-3’
β-Actin	Sense	5’-GTG GGC CGC CCT AGG CAC CAG-3’
	Antisense	5’-GGA GGA AGA GGA TGC GGC AGT-3’

^a^*TNF-α*, tumor necrosis factor-α; *iNOS*, inducible nitric oxide synthase; *COX-2*, cyclooxygenase-2; *IL-1β*, interleukin-1β; *IL-6*, interleukin-6.

**Table 2:** Tolerance of LAB strains under artificial gastric acid and bile salt conditions.

LAB strains	*L. rhamnosus* GG	*L. plantarum* KU15149	*L. brevis* KU15176
Tolerance to artificial gastric condition			
Initial cell number	8.31 ± 0.09b	8.40 ± 0.10a	8.28 ± 0.03c
0.3% pepsin, pH 2.5, 3h	8.25 ± 0.10b	8.33 ± 0.06a	8.07 ± 0.13c
Tolerance to artificial bile condition			
Initial cell number	8.31 ± 0.09b	8.40 ± 0.10a	8.28 ± 0.03c
0.3% oxgall, 24 h	8.55 ± 0.02a	6.87 ± 0.07c	7.36 ± 0.16b

All values are expressed as the mean ± standard deviation. Values with different letters in the same row indicate significant differences for each characteristic (*p* < 0.05).

**Table 3:** Enzymatic activities of LAB strains according to the API ZYM kit.

Enzyme	*L. rhamnosus* GG	*L. plantarum* KU15149	*L. brevis* KU15176
Control	0^a^	0	0
Alkaline phosphatase	0	0	1
Esterase	2	0	2
Esterase lipase	1	1	2
Lipase	0	0	1
Leucine arylamidase	3	3	5
Valine arylamidase	3	2	4
Crystine-arylamidase	0	1	2
Trypsin	0	0	0
α-Chymotrypsin	0	0	0
Acid phosphatase	1	1	2
Naphthol-AS-BI-phosphohydrolase	2	1	2
α-Galactosidase	0	0	1
β-Galactosidase	1	5	5
β-Glucuronidase	0	0	0
α-Glucosidase	0	0	1
β-Glucosidase	1	4	4
N-Acetyl-β-glucosaminidase	0	3	5
α-Mannosidase	0	0	0
α-Fucosidase	0	0	0

0^a^, 0 nmol; 1, 5 nmol; 2, 10 nmol; 3, 20 nmol; 4, 30 nmol; 5, ≥ 40 nmol.

**Table 4:** Antibiotic sensitivities of LAB strains.

Antibiotics	*L. rhamnosus* GG	*L. plantarum* KU15149	*L. brevis* KU15176
Ampicillin (10 mg)	S	S	S
Gentamycin (10 mg)	R	R	R
Kanamycin (30 mg)	R	R	R
Streptomycin (10 mg)	R	R	R
Tetracycline (30 mg)	S	R	S
Ciprofloxacin (30 mg)	R	R	R
Chloramphenicol (30 mg)	S	I	S
Doxycycline (30 mg)	S	S	S

Resistance was evaluated according to the CLSI breakpoints (CLSI, 2012).

S, Susceptible; I, intermediate; R, resistant.
